# Distinguishing Amyloid β-Protein in a Mouse Model of Alzheimer’s Disease by Label-Free Vibrational Imaging

**DOI:** 10.3390/bios11100365

**Published:** 2021-09-30

**Authors:** Shaowei Li, Ziyi Luo, Renlong Zhang, Hao Xu, Ting Zhou, Liwei Liu, Junle Qu

**Affiliations:** Key Laboratory of Optoelectronic Devices and Systems of Guangdong Province and the Ministry of Education, College of Physics and Optoelectronic Engineering, Shenzhen University, Shenzhen 518000, China; lisw@szu.edu.cn (S.L.); luoziyi2020@email.szu.edu.cn (Z.L.); zhangrenlong2020@email.szu.edu.cn (R.Z.); hxuhao@szu.edu.cn (H.X.); worserobot@gmail.com (T.Z.); liulw@szu.edu.cn (L.L.)

**Keywords:** Raman scattering, nonlinear optical microscopy, Alzheimer’s disease, amyloid

## Abstract

Due to the increase in the average age of humans, Alzheimer’s disease (AD) has become one of the disorders with the highest incidence worldwide. Abnormal amyloid β protein (Aβ) accumulation is believed to be the most common cause of AD. Therefore, distinguishing the lesion areas can provide clues for AD diagnosis. Here, we present an optical spectroscopy and imaging approach based on coherent anti-Stokes Raman scattering (CARS). Label-free vibrational imaging of Aβ in a mouse model of AD was performed to distinguish the lesion areas by studying the spectra of regions with and without Aβ plaques. Raman spectra in Aβ and non-Aβ regions exhibited a specific difference in the intensity ratio of the wave peaks detected at 2850 and 2930 cm^−1^. In the non-Aβ region, the ratio of the peak intensity at 2850 cm^−1^ to that at 2930 cm^−1^ was approximately 1, whereas that in the Aβ region was 0.8. This label-free vibrational imaging may provide a new method for the clinical diagnosis and basic research of AD.

## 1. Introduction

Dementia is a progressive neurodegenerative disease characterized by a decline in memory, thinking, learning, and cognitive functions. Alzheimer’s disease (AD) is the most common form of dementia, accounting for approximately 60–70% of the cases. The pathological characteristic of AD is the abnormal deposition of the amyloid β protein (Aβ) in the cortex and hippocampus. However, how Aβ plaques form and how they affect the nervous system remain unclear. The current mainstream hypothesis regarding the pathogenic mechanism of Aβ is that the metabolism of the amyloid precursor protein (APP) and the subsequent condensation of Aβ are the major events driving AD. High levels of Aβ subsequently lead to a series of downstream pathological events, including the production of large intracellular neurofibrillary tangle (NFT) deposits, inflammation, oxidative stress, excitotoxicity, loss of synaptic connections, and cell death, which contribute to the clinical manifestations of AD [1,2,3,4]. Moreover, the underlying pathology of AD begins 10–20 years before the clinical symptoms appear [5]. Therefore, direct imaging of Aβ is of great significance in the diagnosis and prevention of AD. Several techniques have been developed to image Aβ plaques. For example, positron emission tomography (PET) can be used to clinically image Aβ deposition in vivo using various Aβ tracers, such as 11 C-labeled Pittsburgh compound-B [6,7]. In addition, magnetic resonance imaging (MRI) technology can also be used clinically to achieve non-invasive imaging of single plaques in vivo [8]. However, MRI is limited by its low spatial resolution, and PET relies on tracers and cannot achieve label-free imaging. In addition, several studies have shown that lipid metabolism in the brain of patients with AD is closely related to the formation of Aβ plaques [9,10].

Coherent anti-Stokes Raman scattering (CARS) microscopy is an emerging label-free vibrational imaging with chemical selection that has been widely applied in biology and medical research [11,12,13,14,15,16,17]. Because CARS can obtain spectral information while achieving unlabeled imaging, it is highly suitable for studying lipid metabolism and lipid distribution in the brain as well as for the identification of abnormal accumulation of Aβ in AD.

In this paper, we present an optical spectroscopy and imaging approach that is based on CARS imaging of lipid distribution by probing the intrinsic molecular vibrations. These vibrations allow the distinction of Aβ regions from non-Aβ regions based on the differences in the intensity ratio between 2850 and 2930 cm^−1^ in the CARS spectrum.

## 2. Materials and Methods

### 2.1. The CARS Microscope

**Figure 1:**
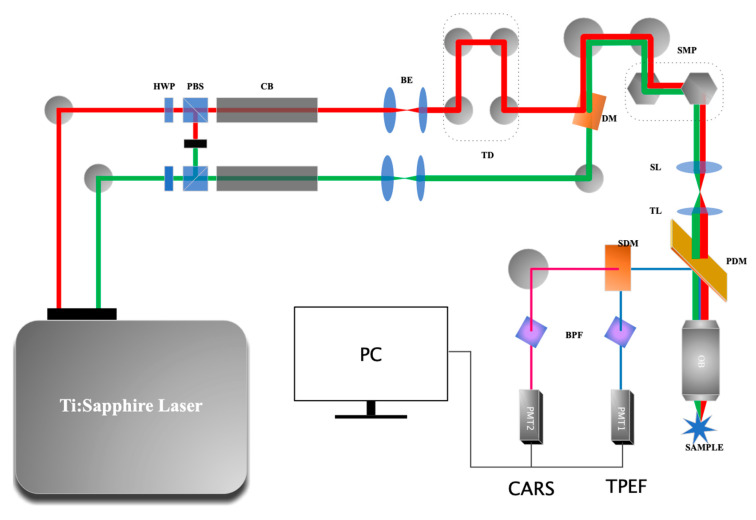
Schematic diagram of the CARS system. Ti: Sapphire Laser, titanium-doped sapphire solid-state laser with two outputs; HWP, half-wave plate; PBS, polarization beam splitter; CB, chirped block; BE, beam expander; TD, time delay; DM, dichroic mirror; SMP, scanning mirror pairs; SL, scan lens; TL, tube lens; PDM, primary dichroic mirror; OB, objective lens; SDM, secondary dichroic mirror; BPF, band-pass filter; PMT, photomultiplier.

Figure 1 provides a diagrammatic representation of the CARS microscope. The light source is a femtosecond (fs) laser (Chameleon Discovery, Coherent Inc.) with two outputs. One output generates a 1040 nm fs laser pulse (pulse width of 100 fs) with a repetition rate of 80 MHz, serving as the Stokes beam for the CARS imaging. The other output is tunable from 660 to 1320 nm (pulse width of 100 fs) and is used as the pump beam for the CARS imaging. We tuned the pump beam to 800 nm (Δω = 2850 cm^−1^) to match the CH_2_ vibration in lipids. The Stokes beam (1040 nm) was collinearly combined with the pump beam (800 nm) through a dichroic mirror and delivered to an upright laser scanning confocal microscope (MPM-SCAN4, Thorlabs Inc., America). The fs pump and Stokes laser beams were chirped using SF-57 glass rods with a length of 40.5 and 54 cm to generate a 2.0 picosecond (ps) pump beam and a 1.8 ps Stokes beam for hyperspectral CARS imaging, respectively, before they were combined with a dichroic mirror. The Raman shift differences between the pump and Stokes beams were scanned by controlling the time delay between the pump and Stokes pulses. A long-pass (680 nm) primary dichroic mirror was used to reflect the signal. Another long-pass (570 nm) secondary dichroic mirror was used to separate the signal to the CARS channel at 650 nm and to the two-photon excited fluorescence (TPEF) channel. After the application of a band-pass filter (650/10 nm, Thorlabs Inc., America), the CARS signal was detected using a photomultiplier tube (H7422-50, Hamamatsu photonics) and the TPEF signal was detected using another photomultiplier tube (H7422-40, Hamamatsu photonics). For CARS imaging, the output power of the pump light is ~50 mW, and for Stokes light, it is ~100 mW. The system was calibrated systematically. The depth of penetration is about 200 μm, which is measured in live imaging, and the field of view under a 20X water immersion objective is 810 × 810 μm. The spatial resolution is about 485 nm, and the spectral resolution is 23 cm^−1^.

### 2.2. Sample Preparation

Twelve-month-old transgenic mice (APP/PS1) were obtained from the Medical Animal Laboratory Center of Guangdong Province (permit number 44007200079864). All experiments were performed with approval of the Medical Department of Shenzhen University. The mice were euthanized by an intraperitoneal injection of 1% sodium pentobarbital (50 mg/kg, Sigma-Aldrich). Subsequently, 0.9% saline and 4% paraformaldehyde were perfused transcranially and chilled to 4 °C. Then, the brain was removed and fixed at 4 °C in 4% paraformaldehyde for 24 h, followed by staining with methoxy-X04(4,4′-[(2-methoxy-1,4-phenylene)di-(1E)-2,1-ethenediyl]bisphenol) (Xcess Biosciences Inc., CA 92109) at 10 µmol/L and paraffin embedding. A rotary microtome was used to cut the brain into five contiguous sections with a thickness of 6 µm.

## 3. Results

**Figure 2:**
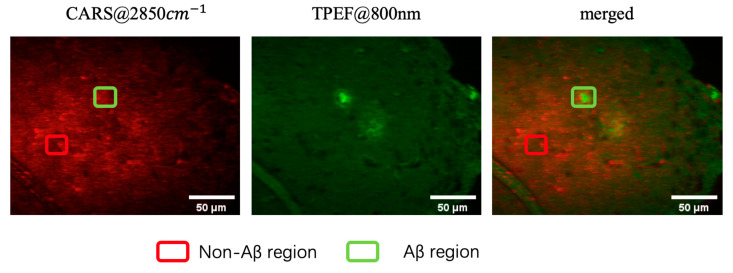
CARS and TPEF images of brain tissue from a mouse model of AD: (**Left**) CARS image acquired at 2850 cm^−1^; (**middle**) TPEF image excited at 800 nm; (**right**) merged image. The red and green boxes represent the non-Aβ and Aβ regions, as indicated by the TPEF image shown in the middle panel. Scale bar, 50 μm.

In this study, Aβ plaques in the brain slice of APP/PS1 transgenic mice were, first, stained by a fluorescent amyloid-β probe, methoxy-X04 [18,19,20,21], which emits strong TPEF. Then, the sample was imaged using TPEF and CARS, and the images were presented in green and red pseudo colors, respectively. The TPEF, CARS, and their merged images are shown in Figure 2. In this study, 20 samples from five different AD mouse are tested, with each mouse providing four samples. The imaging area was located in the cortex of the mouse brain. Lipid droplets in the brain tissue are excited by the CARS process. A prominent blood vessel can be observed in the lower-left area. However, the Aβ plaques were not detected in the CARS image. The middle panel of Figure 2 corresponds to the TPEF image of a brain tissue slice stained with methoxy-X04. Two Aβ plaques are clearly visible, one of which is not in the current focal plane. The merged image of TPEF and CARS (right panel in Figure 2) confirmed the location of the non-Aβ and Aβ regions.

CARS imaging generates contrast by probing the vibrational resonances in molecular bonds. In Raman spectroscopy, the spectrum is divided into three parts: the “fingerprint region,” from 400 to 1500 cm^−1^; the “silence region,” from 1500 to 2700 cm^−1^; the “C–H region,” from 2800 to 3100 cm^−1^. Most vibrational spectroscopy and microscopy approaches focus on the “C–H region” because the signal is stronger than that in the other regions. The C–H region contains strong CH_2_ and CH_3_ symmetrical and asymmetrical stretching modes. Lipids in each molecule typically contain long acyl chains with many C–H bonds. The analysis of the C–H region from 2800 to 3100 cm^−1^ allows the differentiation of molecular markers, such as lipids and proteins, based on the distinct ratio of the methylene CH_2_ to the methyl CH_3_ group. Moreover, the mouse brain tissue is rich in lipids and proteins that can produce strong CH_2_ and CH_3_ molecular vibration signals. The key aspect of multispectral CARS imaging is the acquisition of both highly spatially and spectrally resolved CARS images for subsequent analysis [22,23].

**Figure 3:**
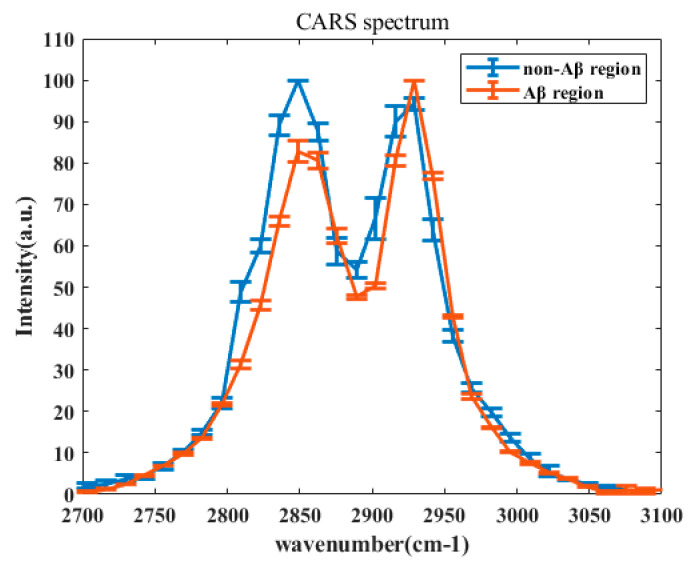
Comparison of typical normalized CARS spectra of the non-Aβ region (blue line) and Aβ region (red line) in the C–H stretching vibration region. Both spectra exhibited two peaks at 2850 and 2930 cm^−1^. The standard errors are indicated by the horizontal solid lines.

**Figure 4:**
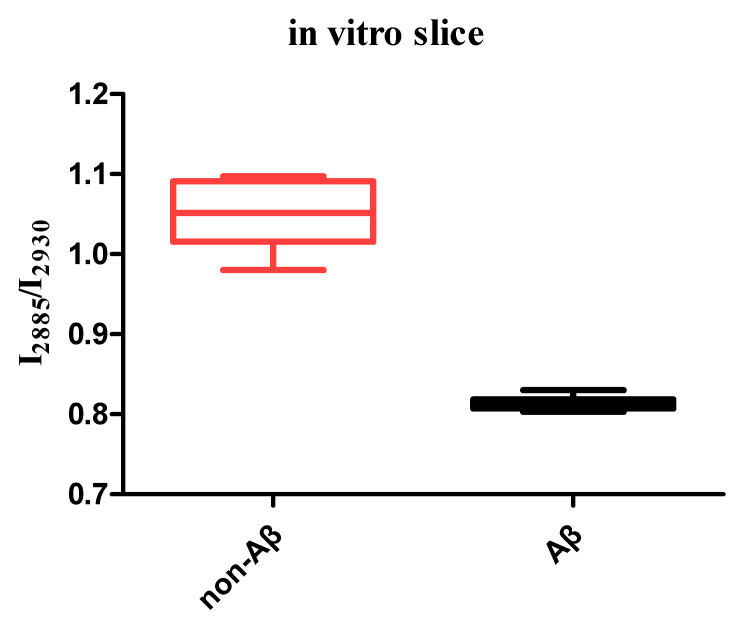
Standard deviation of I_2850_/I_2930_ in the non-Aβ and Aβ regions. Each region contained 20 sets of data. Each set of data was collected from different slices in vitro.

Next, we acquired and analyzed the CARS spectra of the mouse brain tissue in the non-Aβ and Aβ regions. To quantitatively analyze the differences in the spectra of the Aβ region (red line) and non-Aβ region (blue line), we normalized all data. The statistical results obtained after normalization are shown in Figure 3. The two areas exhibited peaks at 2850 and 2930 cm^−1^, respectively, corresponding to the vibrational mode of the methylene CH_2_ and methyl CH_3_ groups. Interestingly, we found that the intensity ratios between the peak at 2850 cm^−1^ and the one at 2930 cm^−1^ in the non-Aβ and Aβ region were quite different, as shown in Figure 4. In the non-Aβ region, the peak intensity at 2850 cm^−1^ was always as high as that at 2930 cm^−1^. However, in the Aβ region, the peak intensity at 2850 cm^−1^ was about four-fifths as strong as that at 2930 cm^−1^, indicating that the symmetric stretching vibrations of CH_2_ in the Aβ-enrichment region were suppressed. This observation suggests that the ratio of lipids to proteins in the Aβ-rich region is lower than that in the non-Aβ-rich region. According to the results of previous research, abnormal lipid metabolism affects the metabolism and deposition of Aβ, which leads to a series of negative effects and ultimately affects the pathogenesis of AD [9,10,24,25,26,27,28,29,30,31,32].

Previous studies have reported the relevance of lipids and Aβ proteins in AD based on coherent Raman scattering microscopy [33,34,35]. Lee et al. [34] studied AD brain samples using multimodal, multiphoton, nonlinear optical micro-spectroscopy. However, the objective of CARS imaging and spectra is γ-aminobutyric acid (GABA), which is a neurotransmitter that has been reported in many research papers, rather than the Aβ protein itself. Ji et al. [33] reported that the Raman shift between normal proteins and the Aβ protein is about 10 cm^−1^; based on this blue shift, his team used stimulated Raman scattering microscopy to image the amyloid plaques in the brain tissue of an AD mouse model. This method requires a coherent Raman scattering microscope with a very high spectral resolution. Moreover, his team mainly studied the spectrum of the amide I band, which is located in the fingerprint region.

**Figure 5:**
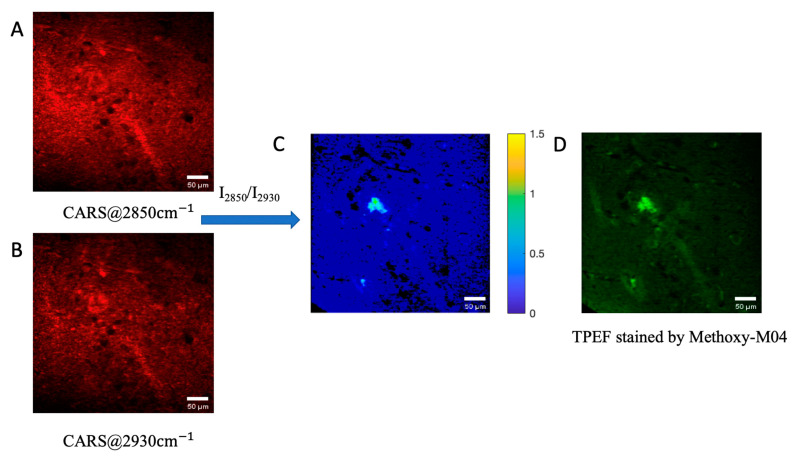
Workflow that can be used to verify the feasibility of distinguishing non-Aβ region from Aβ region in the AD brain tissue without labeling. Individual CARS images are shown: (**A**) 2850 cm^−1^; (**B**) 2930 cm^−1^; (**C**) the result of the image in A divided by the image in B, pseudo color in (**C**) stands for the peak ratio of each pixel. We can find an unusual area in the middle which could be regarded as the Aβ region; (**D**) the TPEF image stained by Methoxy-X04, which is used to locate the Aβ plaques and the Aβ region.

Finally, to verify the feasibility of this method, another set of data was analyzed. According to previous results, a home-built MATLAB procedure was used to calculate and process the hyperspectral CARS data; the results of this analysis are shown in Figure 5. The intensity of each pixel in the CARS image at 2850 cm^−1^ was divided by the corresponding CARS image at 2930 cm^−1^, and the result was shown using pseudo color (middle panel of Figure 5). As compared with the TPEF image of the brain slice stained with methoxy-X04, it is apparent that this method can be used to distinguish the non-Aβ region from the Aβ region in the AD brain tissue without labeling.

## 4. Conclusions

In this study, we developed an optical spectroscopy and imaging approach based on CARS imaging of the Aβ protein to distinguish the non-Aβ region from the Aβ region in the brain tissue of an AD mouse model.

Hyperspectral CARS images were obtained from spectral-focusing-based CARS microscopy. The spectrum of the C–H region contained strong CH_2_ and CH_3_ symmetrical and asymmetrical stretching modes, which can be used as a tool to analyze the lipid and protein components of biological tissues. Specifically, the ratio between the CH_2_ vibration mode at 2850 cm^−1^ and the CH_3_ vibration mode at 2930 cm^−1^ could be used to characterize the lipid or protein components of biological tissues. Moreover, Aβ plaques in the cerebral cortex could be imaged without labeling, and the imaging results are similar to those obtained by labeling with the two-photon dye methoxy-X04. We found that this ratio was about 0.8 in the region that contained the Aβ protein, which was confirmed by TPEF images stained by methoxy-X04. In contrast, the average ratio was about 1.0 in the non-Aβ region. Therefore, this approach is expected to provide a new method for the clinical diagnosis and basic research of AD.

There are two future directions for this research. On the one hand, the Raman spectral characteristics of amyloid in human brain slices should be studied and compared with the spectra in mice. The difficulty of this work lies in the fact that the components of human brain are much more complex than the mouse model, requiring further research. On the other hand, the Raman spectroscopic properties of amyloid in living mice should be investigated, extending the study from in vitro to in vivo. Another interesting future development of this method should be the use of various polynomial fitting methods to analyze the total contribution of CH_2_, CH_3_, and non-resonant background in the spectrum of the C–H region. We except that the results would reveal greater details of lipid metabolism, especially cholesterol metabolism, in the AD brain tissue.

## Data Availability

Not applicable.
